# Trastuzumab-induced human cardiomyocyte damage through the Notch2/JAK2/STAT3 pathway

**DOI:** 10.1016/j.clinsp.2023.100268

**Published:** 2023-08-09

**Authors:** Zhenbo Su, Siyao Liu, Yinggang Zou, Liang Shan, Miao Yu, Shishun Xie, Xiangjun Li, Ying Jin

**Affiliations:** a Department of Anesthesiology, China-Japan Union Hospital of Jilin University, Changchun, China; b Department of Experimental Pharmacology and Toxicology, School of pharmaceutical science, Jilin University, Changchun, China; c Reproductive Medical Center, Department of Obstetrics and Gynecology, The Second Hospital of Jilin University, Changchun, China; d Department of Ultrasound, China-Japan Union Hospital of Jilin University, Changchun, China

**Keywords:** Trastuzumab, Human cardiomyocyte toxicity, Notch2, Cell apoptosis

## Abstract

**Objective:**

Trastuzumab is the preferred drug for the treatment of breast cancer. However, research on the cellular mechanisms of trastuzumab's potential cardiotoxicity is insufficient. The purpose of this study was to explore the toxic effects and potential mechanism of action of trastuzumab on cardiomyocytes.

**Method:**

Human Cardiomyocyte (HCM) viability was assessed using the MTT method. HCM apoptosis was detected using the Hoechst33342/PI Fluorescent staining. The LDH and CK activities of the cell were measured using commercially available LDH and CK assay kits. The expression levels of Notch2, JAK2, STAT3, cleaved caspase 3, bax, and bcl 2 in HCMs were detected using western blotting.

**Results:**

The results showed that 250 mg/L trastuzumab induced cardiomyocyte injury and apoptosis, inhibited viability, activated the Notch2 receptor, and inhibited JAK2/STAT3 expression in HCM. Inhibition of Notch2 expression in HCM by targeted siNotch2 transfection reversed the trastuzumab-induced injury and apoptosis, and the expression of JAK2/STAT3 returned to normal levels.

**Conclusions:**

Trastuzumab induces Notch2 expression by inhibiting the JAK2/STAT3 pathway of HCMs, promotes cell apoptosis, and causes cardiomyocyteinjury. Notch2 may be a potential target of trastuzumab-inducedmyocardial injury. This experiment reveals the mechanism of trastuzumab-induced cardiotoxicity, providing a theoretical basis for the application of trastuzumab.

## Highlights

• Notch2 is important in trastuzumab-induced cardiomyocytes apoptosis through JAK2/STAT3 pathway.
• Trastuzumab promotes cell apoptosis and causes cardiomyocyte injury.
• Notch2 may be a potential target of trastuzumab-induced myocardial injury.

## Introduction

Trastuzumab, a monoclonal antibody belonging to the family of small-molecule tyrosine kinase inhibitors, has been approved for the treatment of Human Epidermal growth factor Receptor 2 (HER2)-positive breast cancer. Clinically, trastuzumab can prolong Progression-Free Survival (PFS) and Overall Survival (OS). However, some studies have reported that women with breast cancer have a higher risk of cardiovascular disease after trastuzumab treatment, including left ventricular dysfunction, congestive heart failure, conduction and rhythm disturbances, acute coronary syndrome, hypertension, pericardial pathology, vasospasm, and dilated cardiomyopathy;^1^ the risk of death from these cardiovascular diseases exceeds that of cancer. Therefore, more attention should be paid to the risk of cardiovascular disease when using trastuzumab in the treatment of breast cancer.^2^,^3^ The mechanism of cardiotoxicity induced by trastuzumab is still unclear but may be related to the inhibition of HER2/NRG1 dimer formation,^4^ activation of the caspase 3/7 pathway, which induces apoptosis and oxidative stress in cardiomyocytes,^5^,^6^ or inhibition of autophagy in cardiomyocytes.^7^

The Notch signaling pathway is a highly conserved signaling pathway in biological evolution that consists of Notch ligands (DSL proteins), Notch receptors, and DNA binding proteins (CSLs). Signals exchanged between neighboring cells through the Notch receptor can amplify and consolidate molecular differences, eventually dictating cell fates.^8^ Evidence indicates that the Notch signaling pathway plays an important role in tumorigenesis and tumor progression. Research has found that the Notch signaling pathway is abnormally activated in breast cancer, and overexpression or abnormal expression of its receptors and ligands is associated with the progression of breast cancer.^9^,^10^

Janus Kinases (JAKs) belongs to a family of non-receptor protein tyrosine kinases, including JAK1-3 and Tyrosine Kinase-2 (TYK2), both of which bind to cytokine receptors in the cytoplasmic region.^11^ Signal Transducer and Activator of Transcription (STAT) family members can be activated in many cells and are also considered oncogenes that have anti-apoptotic effects once activated.^12^ The JAK/STAT pathway is one of the most important signaling pathways related to the functions of various growth factors and cytokines; it transmits information from extracellular sources, especially various cytokines, and regulates DNA transcription and gene expression, ultimately regulating the proliferation, differentiation, apoptosis, and immunity activity of target cells.^13^,^14^

Evidence shows that Notch activity can be regulated by the JAK/STAT pathway. For example, the presence of inflammatory cytokines that activate transmembrane Notch-1 receptors is regulated by STAT-3 phosphorylation.^15^ The effects of leptin on the inhibition of breast cancer proliferation and migration are also induced by activating JAK/STAT3 and inducing Notch expression.^16^ Huang et al.^17^ suggested that MK-induced cross-talk of Notch2/JAK2/STAT3 signaling pathways can regulate cell plasticity and motility, contributing to the EMT and later stages of tumorigenesis.

In this study, the authors aimed to determine the possible relationship between trastuzumab-induced cardiotoxicity and the Notch2/JAK2/STAT3 pathway.

## Materials & methods

### Materials

Trastuzumab for injection (Herceptin) was purchased from Shanghai Roche Pharmaceutical Co., Ltd. (S20110007). Trastuzumab is a white to pale yellow lyophilized powder (440 mg), and the vehicle for injection is 20 mL sterile water containing 1.1% benzyl alcohol.

### Cell culture

Human Cardiomyocytes (HCMs) were maintained in our laboratory. After thawing, HCMs were cultured regularly in DMEM (C11995500BT; Gibco, Invitrogen) containing 10% fetal bovine serum (04-001-1A; Biological Industries, Israel) and 1% penicillin-streptomycin dual antibody (60162ES76; Yeasen Biotechnology (Shanghai) Co, Ltd., Shanghai, China) at 37°C with 5% CO_2_.

### Cell viability and proliferation assay

HCM viability was assessed using the MTT method. HCMs in the logarithmic growth phase were seeded in 96-well plates at a density of 5 × 10^3^ cells/well. The cells were incubated with media containing different concentrations of trastuzumab. After 24h, 20 µL MTT (0.5 mg/mL) was added to each well. The supernatant was removed after incubation for 4h at 37°C, and 150 µL DMSO was added to dissolve the precipitate at room temperature. The absorbance at 570 nm was read using a microplate reader, and the absorbance values of cells in each group were compared.

### Apoptotic cell staining

HCMs were seeded into six-well plates at a density of 5 × 10^4^ cells/well and cultured in conditioned media for 24h. The culture medium was then discarded, and the cells were washed with PBS three times. The cell staining buffer was directly added to the cells, followed sequentially by Hoechst 33342 and PI staining solutions, and incubated at 4°C for 15 min. The staining was observed with a fluorescence microscope (40744ES60, Yeasen Biotechnology [Shanghai] Co., Ltd.).

### CK and LDH activity detection

The LDH and CK activities of the cell were measured from the cell culture supernatant using commercially available LDH (A020) and CK assay kits (A032; Nanjing Jiancheng Bioengineering Institute) to assess cell damage. According to the manufacturer's instructions, the culture supernatant of the HCMs was collected, kit reagents were added, and mixed well. After incubation for 5 min at room temperature, the absorbance value at 450 nm was measured using an enzyme labeling instrument.

### Western blot analysis

Cells were lysed in Radioimmunoprecipitation Assay (RIPA) buffer, and the supernatant was collected by centrifugation at 12,000 rpm for 15 min. The protein concentration was determined using the BCA method. After adjusting the concentration, the protein was denatured in 5 × sodium dodecyl sulfate-polyacrylamide gel electrophoresis (SDS-PAGE) Protein Loading Buffer at 95°C for 5 min and stored at -20°C for later use.

Protein samples were transferred onto Polyvinylidene Difluoride (PVDF) membranes after SDS-PAGE. PVDF membranes were incubated in TBST containing 5% non-fat milk powder for 1h at room temperature. After fully washing the PVDF membrane with TBST, primary antibodies were added and incubated overnight at 4°C. The PVDF membrane was washed three times with TBST for 15 min before adding the HRP-conjugated secondary antibody and incubating the membrane for 1h at room temperature. The membrane was washed a final time in TBST and reacted with an ECL developer. The antibodies used are listed in Table 1.

**Table 1:** Antibodies used in this study.

Antibody	Dilutions	Source	Company
Primary antibodies			
β-actin	1:5000	Mouse	ZENBIO, 8F10
Notch2	1:1000	Rabbit	ABclonal, A0560
p-STAT3	1:2000	Rabbit	Affinity, AF3295
STAT3	1:2000	Rabbit	Affinity, AF6294
JAK2	1:3000	Rabbit	Abcam, 17670-1-AP
Bax	1:3000	Rabbit	ABclonal, A11550
Bcl2	1:1000	Rabbit	ABclonal, A0208
Cleaved-caspase3	1:2000	Rabbit	ABclonal, A0214
Secondary antibody			
HRP Goat Anti-Rabbit IgG	1:3000	Goat	ZENBIO, 511203
HRP Goat Anti-Mouse IgG	1:3000	Goat	YEASEN, 33201ES60

### Real-time RT PCR

Total RNA was extracted using a total RNA rapid extraction kit (Animal tissue/Cell Total RNA Kit, ZP404; Beijing Zoman Biotechnology), and the concentration and purity OD260 and OD280 values were measured, ensuring the OD260/OD280 was > 1.8. Total RNA was reverse-transcribed into cDNA using Hifair® II 1^st^ Strand cDNA Synthesis Kit (Yeasen Biotechnology [Shanghai] Co., Ltd.). The primers were synthesized by Sangon Biotechnology Company (Shanghai, China) with the following sequences: Notch2 forward 3′-TACAGTTGTCGCTGCTTGCC-5′, Notch2 reverse 3′-GACGAAGGTTTCACAGTGCC-5′, GAPDH forward 3′-CATGAGAAGTATGACAACAGCCT-5′, and GAPDH reverse 3′-AGTCCTTCCACGATACCAAAGT-5′. Notch2 expression was determined by qPCR using the Hieff UNICON® Power qPCR SYBR Green Master Mix (Yeasen Biotechnology [Shanghai] Co., Ltd.) in a 20 μL reaction system. Notch2 cDNA level was normalized to that of GAPDH, and the gene expression was analyzed using the 2^−ΔΔCT^ method.^18^

### siNotch2 system construction

Three Notch2 RNA (notch2-3, notch2-2, and notch2-1) and negative control (NC) oligos were prepared. Following the manufacturer's instructions, transfection was performed when the HCM density reached 60%–80%. RNA oligos were transfected into HCMs in a serum-free medium using GP-transfect-mate transfection reagent and incubated for 4–6h. After transfection, the serum-free medium was replaced with a complete medium. Transfection efficiency was calculated as the ratio of positively fluorescent cells to total cells.

### Statistical analysis

All data are expressed as mean ± Standard Deviation (SD) and were obtained from at least three independent experiments. Statistical analysis was performed using GraphPad Prism version 8.0 (GraphPad Software, USA). One-way ANOVA was used for inter-group comparison, and Tukey's post hoc test was used for pairwise comparison. A p-value < 0.05 was considered statistically significant.

## Results

### Effect of trastuzumab on HCMs

**Fig. 1:**
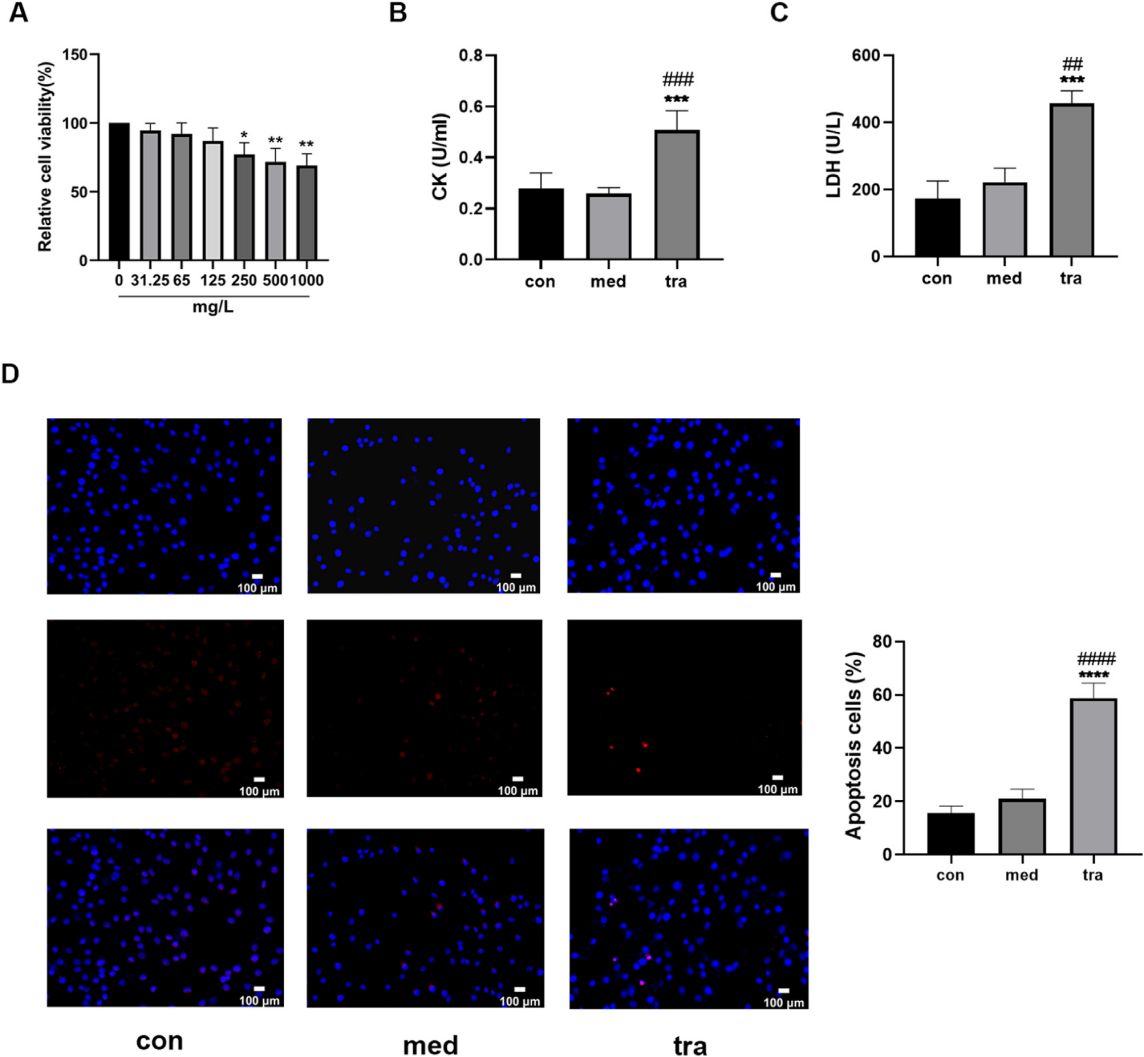
Adverse effects of trastuzumab on HCMs. (A) Comparison of cell viability in the treatment groups. (B, C) Comparison of LDH and CK activities in the treatment groups. (D) Hoechst 33342/PI fluorescence staining results. (E) Comparison of the number of apoptotic cells in the treatment groups. Compared with the control group (con): *p < 0.05, **p < 0.01, ***p < 0.001; Compared with the vehicle group (med): #p < 0.05, ##p < 0.01, ###p < 0.001.

The viability of HCMs decreased significantly when treated with a medium containing 250, 500, or 1000 mg/L trastuzumab for 24h (p < 0.05). A dose-dependent decreasing trend was observed, but no significant differences were found among the three groups (Fig. 1A). Therefore, a concentration of 250 mg/L trastuzumab was used in subsequent experiments.

Assay results showed that the CK and LDH levels in the 250 mg/L trastuzumab-treated group (tra) were significantly increased (p < 0.05) compared with those in the normal control (con) and vehicle groups (med) (Fig. 1 B and C). Hoechst 33342/PI double fluorescent staining was used to assess the level of apoptosis (Fig. 1D); normal cells have both weak red and blue fluorescence, whereas apoptotic cells have weak red fluorescence but strong blue fluorescence. The number of apoptotic cells with double fluorescent staining was approximately 60% in the tra, which was significantly higher than that in the con and med (p < 0.05) (Fig. 1E).

### Effect of trastuzumab on the expression of Notch2, JAK2, STAT3, cleaved caspase 3, bax, and bcl 2 in HCMs

**Fig. 2:**
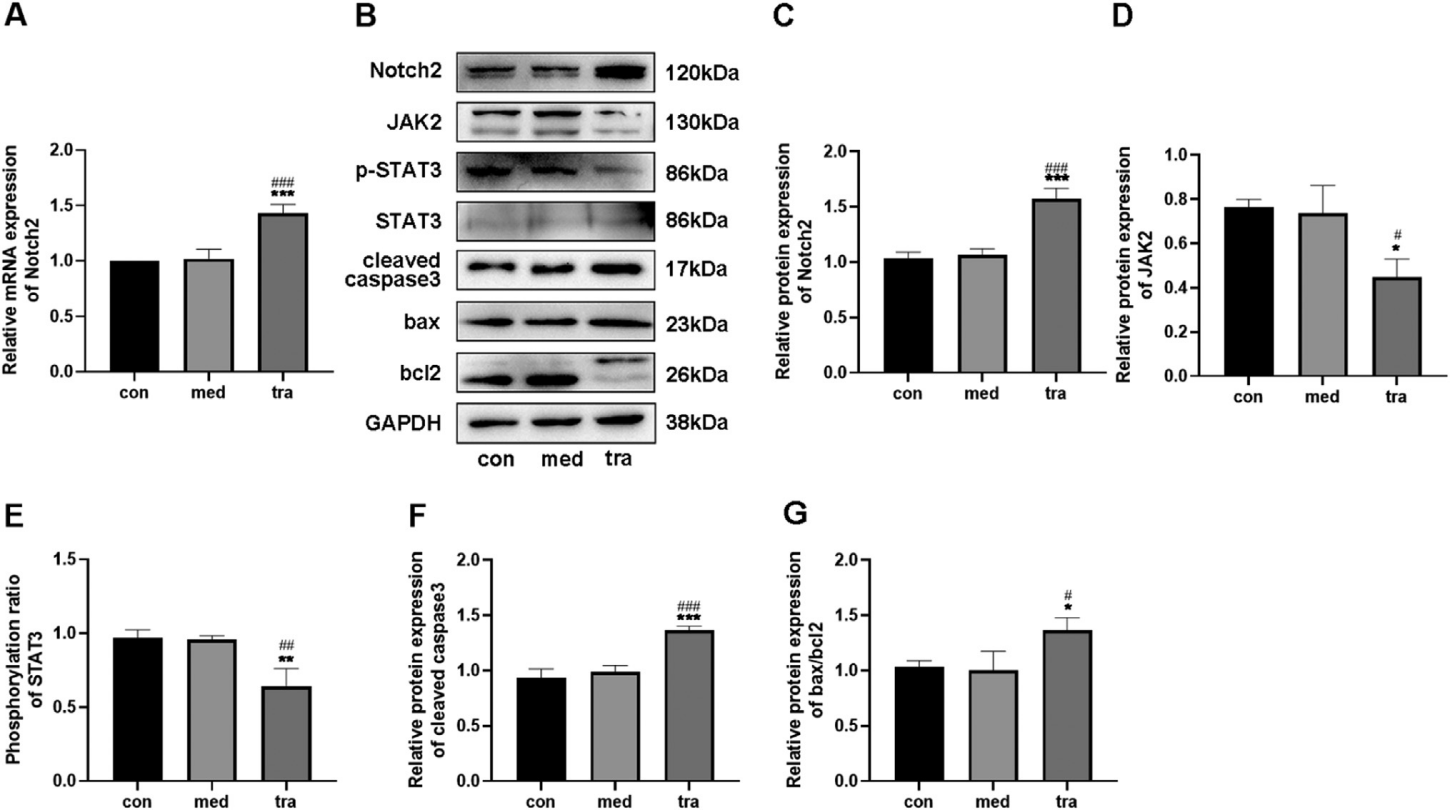
Expression of the Notch2/JAK2/STAT3 pathway and apoptosis-related proteins. (A) Comparison of mRNA expression of Notch2 in the treatment groups. (B) Western blot results in the treatment groups. Analysis of the density of (C) Notch2, (D) JAK2, (E) p-STAT3, STAT3, (F) cleaved caspase3, (G) bax, and bcl2 protein expression in the treatment groups. Compared with the con group: *p < 0.05, **p < 0.01, ***p < 0.001; Compared with the med group: #p < 0.05, ##p < 0.01, ###p < 0.001. Data are presented as mean ± SD (n = 3).

RT-qPCR results showed that the Notch2 mRNA level in the tra was higher than that in the con and med (p < 0.05) (Fig. 2A). Western blotting confirmed that the expression of Notch2 in the tra was significantly increased compared with that in the con and med (Fig. 2 B and C), whereas JAK2 expression and p-STAT3/STAT3 ratio decreased significantly (p < 0.05) (Fig. 2 D and E).

The protein expression of cleaved caspase3, bax, and bcl2 was also detected using western blotting (Fig. 2 F and G). The expression of cleaved caspase3 and bax was significantly higher in the tra than in the con and med, whereas the expression of bcl2 was significantly decreased (p < 0.05). The increased bax/bcl-2 ratio in the tra indicated a pro-apoptotic cell state.

### Results of siNotch2 primer screening

**Fig. 3:**
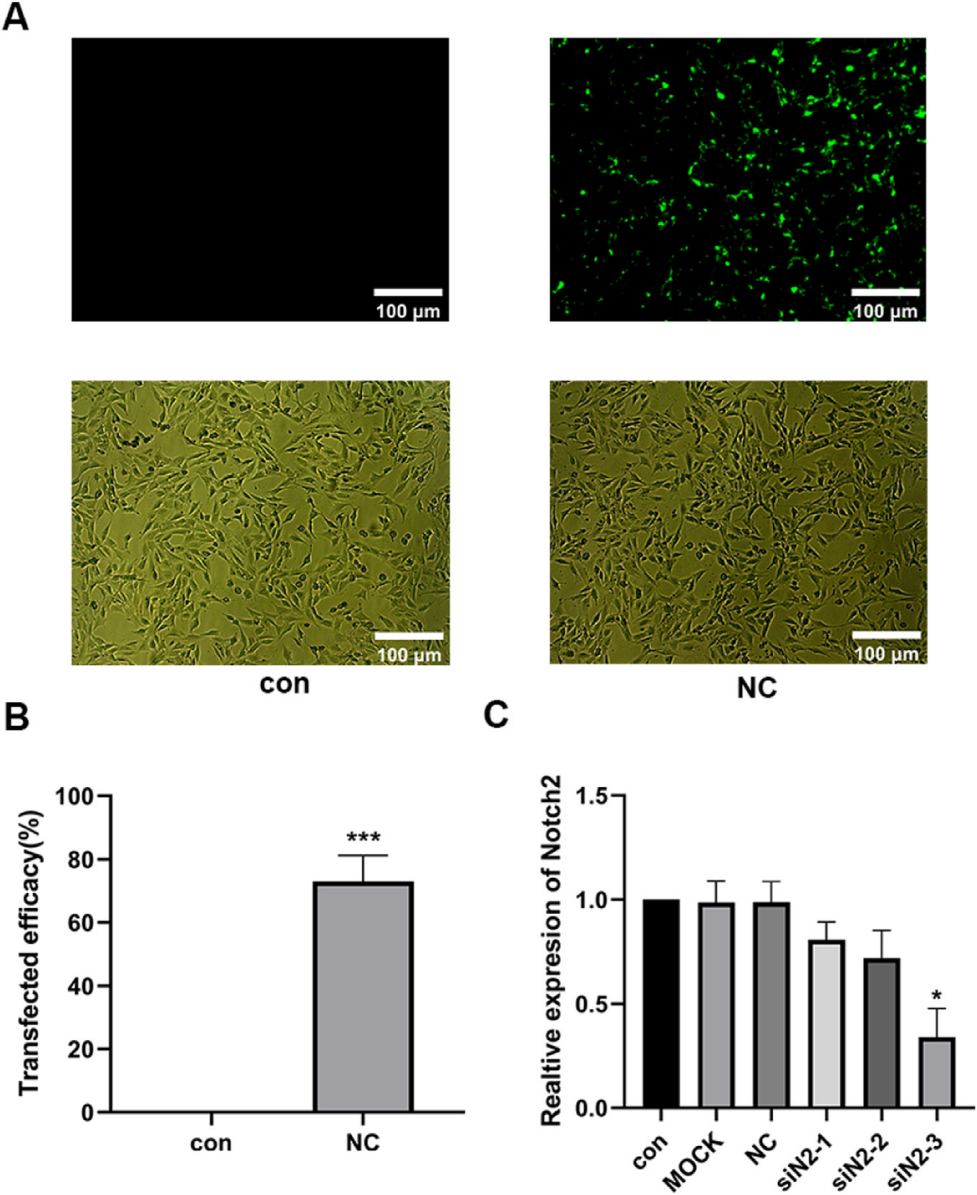
siNotch2 primer screening. (A‒B) Transfection efficiency evaluation for siNotch2. (C) Comparison of Notch2 mRNA expression after transfection with siNotch2. Compared with the con group: *p < 0.05, **p < 0.01, ***p < 0.001. Data are presented as mean ± SD (n = 3).

Three siNotch2 primers were designed based on the Notch2 mRNA sequence (NM_001200001.2), and an irrelevant sequence was used as a negative control. A transfection efficiency of 95% was observed for all primers, and no significant changes in cell morphology were found (Fig. 3 A and B). RT-qPCR was then used to detect the transcription level of Notch2 following siNotch2 transfection. Of the three siNotch2 primer groups, the Notch2 mRNA expression was the lowest after transfection with notch2-3, which was approximately 30% lower than the expression in the con and med (Fig. 3C).

### Trastuzumab induced HCM injury by regulating the Notch2/JAK2/STAT3 axis

**Fig. 4:**
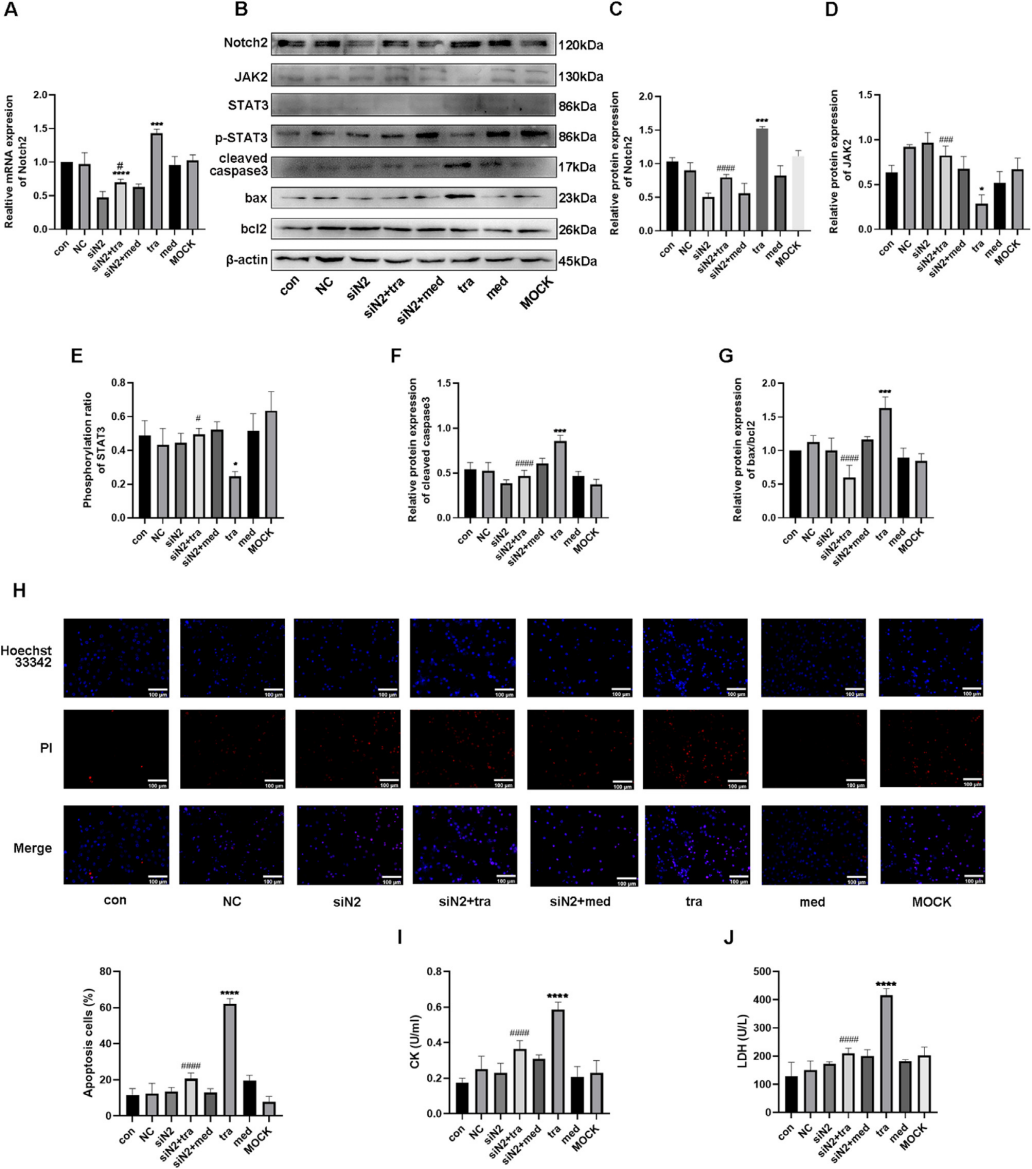
Effects of the Notch2/JAK2/STAT3 axis in trastuzumab-induced HCM injury. (A) Comparison of mRNA expression of Notch2 in the treatment groups. (B) Western blot analysis results in the treatment groups. (C, D, E, F, G) Analysis of the density of Notch2, JAK2, p-STAT3, STAT3, cleaved caspase3, bax, and bcl2 protein expression in the treatment groups. (H) Detection of HCM apoptosis by Hoechst 33342/PI fluorescence. (I, J) Detection of LDH and CK injury indexes in HCM supernatant by microplate method. Compared with the con group: *p < 0.05, **p < 0.01, ***p < 0.001, **** p < 0.0001; The tra compared with the siN2: #p < 0.05, ##p < 0.01, ###p < 0.001, ####p < 0.0001. Data are presented as mean ± SD (n = 3).

As shown in Figure 4A, mRNA expression of Notch2 in the tra increased significantly compared with that in the con and med, whereas it decreased in the siNotch2 group (siN2). Compared with that in the tra, mRNA expression of Notch2 in the siNotch2 + trastuzumab group (siN2+tra) was significantly decreased (p < 0.05). Similarly, the protein expression of Notch2 was significantly increased in the tra and decreased in the siN2 compared with that in the con (Fig. 4 B and C). Moreover, the Notch2 protein expression in both the siN2 and siN2+tra was significantly decreased compared to that in the tra. No significant difference in Notch2 expression was observed between the siN2 and siN2+tra (p < 0.05).

The protein expression of the JAK/STAT pathway was then analyzed. Compared with that in the con, the expression of JAK2 was significantly decreased in the tra, whereas no difference was observed in the siN2 (Fig. 4D). Moreover, the expression of JAK2 in the siN2 and siN2+tra was significantly increased compared with that in the tra. Similar to JAK2 expression, the phosphorylation ratio of STAT3 in the tra was significantly decreased compared with that in the con, whereas no difference was observed in the siN2 (Fig. 4E). The STAT3 phosphorylation ratio in the siN2 and siN2+tra was significantly increased compared with that in the tra. No significant difference in JAK2 expression and STAT3 phosphorylation ratio was observed between the siN2 and siN2+tra (p < 0.05).

Finally, the protein expression of cleaved caspase3, bax, and bcl2 was investigated. As shown in Figure 4F, the expression of cleaved caspase3 in the tra was significantly increased compared with that in the con, whereas no difference was observed in the siN2. Compared with that in the tra, the expression of cleaved caspase3 in the siN2 and siN2+tra was significantly decreased, with no significant difference between the siN2 and siN2+tra (p < 0.05). Similarly, the ratio of bax/bcl2 in the tra was significantly increased compared with that in the con, whereas no difference was observed in the siN2 (Fig. 4G). Compared with that in the tra, the ratio of bax/bcl2 in the siN2 and siN2+tra was significantly decreased, with no significant difference between the siN2 and siN2+tra (p < 0.05). These results suggest that Notch2 is important in trastuzumab-induced cardiomyocyte apoptosis through the JAK2/STAT3 pathway.

Analysis of apoptosis showed that the number of apoptotic cells in the tra was significantly increased compared with that in the con (Fig. 4H). However, no significant difference was observed between the number of apoptotic cells in the siN2 and con. Compared with that in the tra, the number of apoptotic cells in the siN2 and siN2+tra was significantly decreased. No significant difference in apoptotic cells was observed between the siN2 and siN2+tra (p < 0.05).

Similar to the results of the apoptosis assay, the activities of CK and LDH in the tra were significantly increased compared with those in the con, whereas no difference was observed in the siN2 (Fig. 4 I and J). Compared with those in the tra, the CK and LDH activities in the siN2 and siN2+tra were significantly decreased. No significant differences in CK and LDH activities were observed between the siN2 and siN2+tra (p < 0.05).

## Discussion

Although trastuzumab can improve PFS and OS in patients with HER2-positive breast cancer, its accompanying cardiotoxicity limits its application.^19^ The mechanism underlying the effect of trastuzumab on myocardial function and morphology remains unclear. In this study, the authors found that trastuzumab significantly inhibited the proliferative activity of HCMs and stimulated CK and LDH secretion, suggesting that trastuzumab has adverse effects on cardiomyocytes *in vitro*. Increased cleaved caspase3 and bax expression and decreased bcl-2 expression were also observed in the trastuzumab group, which led to an increase in apoptosis, indicating that trastuzumab-induced HCM injury may be related to activation of the bax/bcl2 pathway.

Notch family receptors are transmembrane proteins located on the cell surface that participate in the regulation of cell proliferation^20^ and apoptosis.^21^ Chen et al.^22^ found that LPS can induce cardiomyocyte apoptosis through the Notch signaling pathway. Notch1 activation reduces cardiomyocyte apoptosis after ischemia by regulating bcl-2, bax, and caspase3 expression.^23^ In this study, HCMs treated with trastuzumab for 24h significantly increased both transcriptional and translational levels of Notch2 and cleaved caspase3, the bax/bcl2 ratio, and cardiomyocyte apoptosis. After inhibition of HCM Notch2 expression with siNotch2, these phenomena were significantly reversed, indicating that Notch2 may have an important role in trastuzumab-induced HCM injury.

Previous research demonstrated that cardiomyocyte apoptosis caused by acute myocardial infarction resulted in a decrease in the bcl-2/bax ratio and expression of p-JAK2 and p-STAT3, while the expression of p-JAK2 and p-STAT3 protein increased significantly. After treatment with the protective drug edaravone, the bcl-2/bax ratio was significantly up-regulated, and myocardial apoptosis decreased significantly.^24^ Kabel and Elkhoely.^25^ found that trastuzumab increased serum CK-MB and LDH in mice, which indicated heart injury by down-regulating STAT3 expression, while rosuvastatin and ubiquinone significantly increased STAT3 expression, reversing trastuzumab-induced cardiotoxicity and improving histopathological changes. In this study, the expression of JAK2 and ratio of phosphorylated STAT3 were significantly decreased in HCMs cultured with trastuzumab, whereas inhibiting HCM Notch2 expression increased JAK2 expression and the STAT3 phosphorylation ratio. In addition, the ratio of bax/bcl2 and number of apoptotic cells was significantly reduced. These results indicate that trastuzumab-induced HCM apoptosis is related to the activation of the JAK2/STAT3 pathway.

In summary, the Notch2/JAK2/STAT3 pathway plays an important role in protecting the heart from apoptosis. Trastuzumab induces Notch2 expression by inhibiting the JAK2/STAT3 pathway of HCMs, promotes cell apoptosis, and causes cardiomyocyte injury. Notch2 may, therefore, be a potential target of trastuzumab-induced myocardial injury.

## Funding

This work was supported by the Health Commission of Jilin Province and Jilin Province Department of Finance: Mechanism research and imaging assessment of impairment of cardiac function due to targeted therapy in HER2 positive breast cancer patients, issue number: 3D517EC93430.

## CRediT authorship contribution statement

**Zhenbo Su:** Conceptualization, Data curation, Funding acquisition, Methodology, Writing – original draft, Writing – review & editing. **Siyao Liu:** Conceptualization, Data curation, Funding acquisition, Methodology, Writing – original draft, Writing – review & editing. **Yinggang Zou:** Data curation, Methodology. **Liang Shan:** Data curation, Formal analysis. **Miao Yu:** Data curation, Investigation. **Shishun Xie:** Data curation, Project administration. **Xiangjun Li:** Data curation, Investigation. **Ying Jin:** Conceptualization, Data curation, Formal analysis, Funding acquisition.

## Declaration of Competing Interest

The authors declare no conflicts of interest.

## Appendix Supplementary materials

Image, application 1
