# Supplementary material for: Trastuzumab-induced human cardiomyocyte damage through the Notch2/JAK2/STAT3 pathway
Source: Clinics (Sao Paulo). 2023 Aug 9;78:100268. doi: 10.1016/j.clinsp.2023.100268 (PMC10432602; doi:10.1016/j.clinsp.2023.100268)
Supplement: Supplementary file 1 [file mmc1.pdf]

editage

Editing Certificate

This document certifies that the manuscript listed below has been edited to ensure language and grammar accuracy and is error free in these aspects. The edit was performed by professional editors at Editage, a division of Cactus Communications. The author's core research ideas were not altered in any way during the editing process. The quality of the edit has been guaranteed, with the assumption that our suggested changes have been accepted and the text has not been further altered without the knowledge of our editors.

MANUSCRIPT TITLE

Trastuzumab-induced human cardiomyocyte damage through the Notch2/JAK2/STAT3 pathway

AUTHORS

Zhenbo Su , Siyao Liu , Yinggang Zou, Liang Shan , Miao Yu , Shishun Xie, Xiangjun Li , Ying Jin

ISSUED ON

April 29, 2023

JOB CODE

ANNUO_774

Vikas Narang
Chief Operating Officer - Editage

editage

Editage, a brand of Cactus Communications, offers professional English language editing and publication support services to authors engaged in over 1300 areas of research. Through its community of experienced editors, which includes doctors, engineers, published scientists, and researchers with peer review experience, Editage has successfully helped authors get published in internationally reputed journals. Authors who work with Editage are guaranteed excellent language quality and timely delivery.

GLOBAL :
+1(833) 979-0061 | request@editage.com

CHINA :
400-120-3020或021-6020-9400 |
fabiao@editage.cn

CACTUS

impact.science

researcher.life

lifesciences.cactusglobal.com

editage.com | editage.co.kr | editage.jp | editage.cn | editage.com.br | editage.com.tw | editage.de
